# Ethical Precision in Nanoscale Brain Interfacing

**DOI:** 10.1002/advs.202524255

**Published:** 2026-04-21

**Authors:** Guilherme Wood

**Affiliations:** ^1^ Institute of Psychology University of Graz Graz Austria

**Keywords:** continuous oversight, ethical precision, moral invasiveness, nanoscale brain interfacing, recovery–discovery framework

## Abstract

Recent advances in nanotechnological brain interfacing are transforming precision from a technical achievement into a new form of intimate access to neural and mental life. Nanoscale sensors and stimulators approach synaptic resolution while reducing anatomical invasiveness, yet this same refinement increases moral invasiveness by enabling inferences about states, dispositions, and traits that individuals may not intend to disclose. To clarify the ethical implications of this shift, the recovery–discovery framework is applied, which conceptualizes neurotechnological uses along two continuous dimensions: restoring prior function (recovery) and generating new knowledge or capacities (discovery). At the nanoscale, these orientations become probabilistically entangled: recovery requires unprecedented characterization of neural pathways, while discovery introduces uncertainty and potential perturbation, creating new or unforeseen needs for recovery. This entanglement undermines device‐centric and episodic models of oversight, requiring ethics to become continuous, operation‐sensitive, and context‐aware. Drawing on insights from science and technology studies and safety cultures such as aviation, the article proposes an architecture of ethical precision, including purpose‐bound operation, capability pre‐registration, epistemic firewalls, intimacy indices, reversibility budgets, and ethics telemetry‐to govern intimate neural access with proportionality and accountability. Precision brain interfacing will advance not only through technical refinement but through the precision of the ethical practices.

## Graphical abstract

As brain interfaces approach the nanoscale, precision no longer only measures—it knows, predicts, and potentially reshapes the mind. This work argues that traditional ethics fails under such conditions and proposes a shift toward continuous, operation‐based governance using the recovery–discovery framework to track, constrain, and responsibly steer emerging forms of epistemic power.

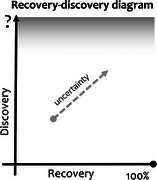

## 1 Introduction: Precision as a New Form of Intimacy

Recent advances in nanotechnological brain interfacing offer an unprecedented degree of access to neural activity [1]. Nanoscale electrodes, molecular sensors, and ultrafine stimulation technologies now approach the spatial and functional resolution of synapses and dendritic compartments [2, 3, 4, 5]. This progress is typically described in terms of precision ‐the refinement of measurement and intervention that improves clinical outcomes, supports closed‐loop neurorehabilitation [6], and deepens our understanding of complex brain dynamics. Yet at these scales, precision is no longer a strictly technical attribute but also an ethical variable [7]. Such forms of access co‐produce not only new modes of measurement but also new ethical conditions, as the technical enactment of precision and the normative stakes of intimate neural access evolve together in unexpected ways [8]. While nanotechnological brain interfacing contributes to decrease physical invasiveness, it may still increase considerably **moral invasiveness** [9, 10]. Moral Invasiveness is the ethical depth of mental access enabled by an interface, independent of anatomical penetration and reflects the potential to reveal, infer, or modulate states considered private or identity‐relevant.

As measurement becomes capable of detecting minute intracellular and synaptic events, the act of observing neural activity acquires a quality traditionally associated with personal and mental privacy: it touches the substrate of subjectivity itself. Conversely, interventions that are physically minimally invasive can still be epistemically invasive, revealing latent states, predictions, or dispositions that individuals never intend to disclose [11, 12]. In this sense, anatomical proximity is replaced by epistemic proximity [13, 14, 15] ‐a novel form of closeness that arises not from physical penetration but from the resolution and inferential power of the interface [16].

This article develops the thesis that such intimate precision destabilizes familiar ethical categories. Drawing on a recovery–discovery framework [17, 18], I argue that nanoscale brain interfacing entangles therapeutic restoration and exploratory investigation in ways that make classical device‐centric and episodic ethical oversight insufficient. Because each enactment of precision constitutes a situated ethical moment [19, 20], appropriate governance must extend beyond fixed procedures to forms of continuous ethical calibration [21]. At the nanometric scale, precision has a moral as well as a technical dimension, requiring ongoing attention to purpose, proportionality, and reversibility: as we approach the brain with greater precision, we must approach the mind with greater care. Ethical analyses of brain–computer interfaces and neurotechnologies consistently emphasize that increased technical capability raises parallel concerns regarding autonomy, dignity, mental privacy, and responsible governance of neural data and interventions [22, 23, 24]. In particular, scholars have noted that the capacity of neurotechnologies to decode or modulate neural signals creates unprecedented forms of access to mental states, making careful ethical oversight and proportional use essential as technological precision advances [25, 26].

## 2 The Meanings of Precision: Technological and Ethical Dimensions

Precision in brain interfacing is often treated as a purely technical property: increased spatial resolution, reduced noise, higher signal fidelity, and more selective stimulation. In this specific sense, precision is synonymous with improved accuracy and minimal invasiveness. Yet technological refinement alone does not capture what nanoscale access actually changes. As interfaces acquire the ability to resolve neural events that are closely tied to perception, intention, affect, or identity, precision becomes a form of epistemic access ‐a way of approaching aspects of a person previously shielded by *biological opacity* [2, 27]. This access is intimate not because it is physically intrusive, but because it enables inferences about states, dispositions, or traits that individuals may not consciously know, let alone choose to reveal. In this sense, the technical enactment of precision and the ethical stakes of intimate neural access are co‐produced: each increment in resolution simultaneously expands the epistemic horizon [28] and alters the normative conditions under which such access must be evaluated.

At this juncture, a second meaning of precision emerges: **ethical precision** [7, 29, 30]. **Ethical precision** concerns the suitability of access relative to purpose, the proportionality of intervention relative to benefit, and the reversibility of any induced changes. **Ethical precision** is thus not about achieving more control or better information, but about responsibly handling them, and ‐because ethical meaning arises within specific contexts‐ about sustaining this calibration as circumstances evolve.

The distinction between technological and **ethical precision** becomes critical because nanoscale interfaces routinely collapse the practical separation between measurement and manipulation (e.g. [31]). When the act of observing neural activity already participates in shaping it, the traditional assumption that precision merely improves therapeutic outcomes no longer holds. The growing relevance of **ethical precision** also exposes the limits of the traditional [32] medical definition of invasiveness [33, 34], which is largely restricted to anatomical disruption ‐how much tissue is penetrated, displaced, or damaged [9, 10]. At the nanoscale, this metric becomes misleading: interfaces may enter the body with a negligible physical footprint while potentially exerting profound epistemic and moral intrusiveness. **Moral invasiveness** refers not to the degree of surgical impact, but to the depth of mental access an interface affords ‐the capacity to reveal, infer, or modulate states that individuals regard as private, identity‐relevant, or not fully under their reflective control [13, 14, 35]. As technological precision reduces bodily harm, it simultaneously increases the granularity with which neural processes tied to subjectivity can be accessed and interpreted. Invasiveness thus becomes a 2D concept: anatomically minimal yet potentially maximal in its ethical gravity. Recognizing this divergence is essential for understanding why nanoscale precision demands new forms of vigilance that cannot be captured by clinical risk classifications alone [8].

## 3 Precision Through the Recovery–Discovery Lens

The ethical significance of nanoscale precision becomes clearer when viewed through a framework for ethical decision making toward neurotechnology: the recovery‐discovery (R/D) framework, which distinguishes two fundamental orientations of neurotechnological practice [17, 18]. *Recovery* aims at restoring functions that have been lost or impaired. Its logic is circular and homeostatic: improvement is measured by how closely a person returns to a familiar and desired state. *Discovery*, by contrast, seeks to generate new knowledge or capacities. Its logic is exploratory and future‐oriented, measured not against a prior norm but by the novelty or insight it produces.

Traditionally, these orientations occupy different normative spaces [17]. Recovery, which is a concept adapted from aviation. Here, the operational envelope describes a defined boundary of safe and permissible system functioning that prevents ethically hazardous excursions unless explicitly justified, documented, and monitored a continuous dimension describing the extent to which a neurotechnology aims to restore prior function or wellbeing, measured against a known and desirable state. Recovery is associated with care, proportionality, and therapeutic intent. Discovery is a continuous dimension capturing the degree to which a neurotechnology generates new knowledge, capacities, or exploratory insights beyond existing baselines. Discovery belongs to the sphere of research, experimentation, and the expansion of understanding. Hitherto, ethical oversight has relied on this separation: what restores and what explores can be governed differently, in part because invasiveness has been defined primarily in anatomical terms, and in part because the pathways through which risks unfold were assumed to be discrete [36].

**FIGURE 1:**
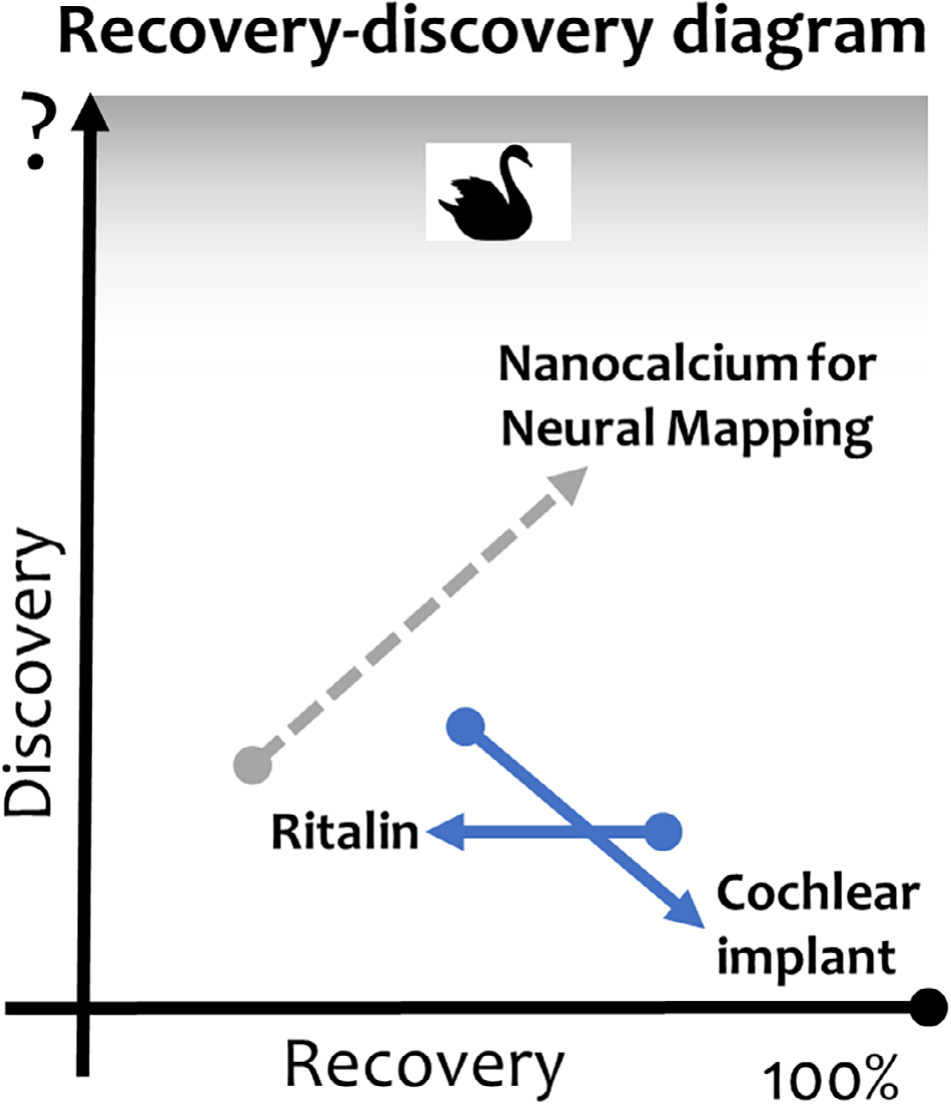
The recovery‐discovery diagram contains two latent and independent dimensions recovery and discovery, which build a coordinate space useful to track uses of neurotechnologies. Three examples of neurotechnologies are depicted. Blue arrows represent trajectories documented by recent scientific literature, while gray ones represent projections. The black swan depicted on the top inhabits regions of the diagram characterized by very high uncertainty and risks.

In the R/D framework, these orientations are not binary categories but continuous dimensions and are represented in the R/D diagram (Figure 1). Any concrete use of a neurotechnology occupies a specific position in a 2D space: it always involves some degree of recovery and some degree of discovery, even if one dominates. The black swan on the top side of the diagram (Figure 1) indicates extreme levels of discovery—a region of very high uncertainty [37] or *“unknown unknowns”*, where the evaluation of risks and disruptive effects using scientific and engineering methods is very challenging [38].

Moreover, the position of a specific technology use is not static. As neural interfaces act, learn, and adapt, their operations may drift across the recovery–discovery plane, moving toward higher discovery (and thus greater uncertainty) or toward greater recovery (and thus higher clinical efficiency). For example (Figure 1), cochlear implants have moved over two decades from a mid‐recovery, moderate‐discovery position toward a predominantly high‐recovery region: improved electrode arrays, signal‐processing strategies, and long‐term outcome data have reduced uncertainty while increasing therapeutic efficacy. By contrast, stimulant medications such as methylphenidate illustrate a different trajectory: as longitudinal evidence accumulates, the recovery coordinate slightly decreases due to diminishing effectiveness over prolonged use, while the discovery coordinate remains largely unchanged, reflecting a stable but modest knowledge gain rather than expanding uncertainty. Figure 1 also depicts an application of nano‐calcium for in vivo neural mapping [39, 40] as a promising example of precision nano‐neurotechnology. Because the depicted trajectory of this neurotechnology is presently only a projection, it is represented as a traced gray arrow. It points toward an increase in recovery (because of promising clinical applications) but also toward an increase in discovery, because of its highly disruptive potential as well as associated risks (including neurotoxicity from reactive oxygen species, blood‐brain barrier disruption, accumulation in brain endothelial cells, which may compromise microcirculation, causing inflammation [40]).

In summary, maturity in technology development efforts lead to a decrease in the risk associated with the use of a specific neurotechnology and consequentially to a reduction in the dimension discovery. Clinical studies leading to the improvement of the same technology typically yield an increase in the dimension recovery [17]. Therefore, ethical evaluation must attend not only to where an operation begins, but to the trajectory it is known or at least expected to follow.

At the nanoscale, this dynamic structure becomes especially salient. The very features that make nanoscale interfaces powerful for recovery‐ultra‐local sensing, high‐bandwidth monitoring, closed‐loop responsiveness are the same features that generate fine‐grained maps of neural dynamics, predictive models of behavior, and insights into mechanisms that were previously opaque [1, 2, 5]. These forms of epistemic access can be anatomically minimal while nevertheless involving substantial ethical intrusion, as discussed above. Conversely, the techniques used for discovery‐single‐neuron recording, synaptic mapping, and molecular tagging are themselves perturbative. They alter the substrate they analyze, and although these alterations may occasionally yield therapeutic benefits, they may also create harms, neutral changes, or mixed outcomes. Discovery operations thus carry forms of intrusion that are not adequately captured by anatomical measures alone.

This dynamic produces a more complex condition of entanglement (e.g., in theranostics, see [41]). This entanglement of discovery and recovery is asymmetrical: every act of recovery requires discovery, because restoring function demands unprecedented characterization of neural pathways; not every act of discovery produces recovery, yet discovery inherently generates uncertainty, and uncertainty carries risk [42]. The deeper the discovery, the greater the likelihood that some form of recovery may become necessary afterward, whether to mitigate unintended alterations, reverse destabilizing perturbations, or address new vulnerabilities created by intimate neural access [43, 44, 45]. In this sense, recovery and discovery remain distinct in purpose, but they become probabilistically interdependent in their consequences, and operations may drift into regions of higher ethical burden as discovery increases [46].

Entanglement does not imply that recovery and discovery collapse into a single category, but that they share operational pipelines and mutually condition each other's ethical landscape. Their methods, data streams, and algorithmic processes converge even as their intentions remain distinct. Each enactment of precision co‐produces a new ethical configuration, as the depth and kind of access afforded by the interface generate shifting conditions of vulnerability, reversibility, and accountability. Ethical evaluation can therefore no longer rely solely on declared purpose or device classification. What matters is the mode in which precision is enacted, the depth and type of invasiveness it entails [36], its position and movement within the recovery–discovery space, the uncertainty it generates, and the proportionality of its effects. This sets the stage for a new kind of ethical scrutiny‐one attentive to trajectories rather than labels, to operations rather than categories, and to uncertainty rather than intention alone.

## 4 Entanglement Forces a Shift to Continuous Ethics

If recovery and discovery become entangled at the nanoscale, the ethical demands of brain interfacing change in a fundamental way. Traditional oversight systems were built around a different technological era, one in which interventions were discrete, purposes were segregated, and the boundary between observation and manipulation could be clearly drawn. State of art neuroethics frameworks already recognized that entanglement dissolves these assumptions [47, 48]. It requires ethics to follow the *operation* rather than the device, and to act *continuously* rather than episodically, because the ethical meaning of an interface is co‐produced with each new enactment of precision and with each movement across the recovery–discovery space [48, 49].

First, nanoscale precision makes the act of measurement inherently participatory. The closer one comes to the neural substrate, the more observation itself becomes a form of modulation. Because discovery at these scales introduces uncertainty, and uncertainty is the generator of risk, the ethical status of an operation cannot be judged solely before it begins. It must be monitored while it is occurring, as informational, physiological, and interpretive effects unfold together, shifting the operation's position in the recovery–discovery plane and potentially creating new needs for recovery. In this context, ethical intrusion can increase even when physical intervention remains minimal [36], adding a dynamic dimension to risk, which traditional classifications cannot capture.

Second, closed‐loop and adaptive systems continuously adjust their parameters in response to ongoing data. Their behavior is not fixed at the time of deployment but evolves as models update, filters recalibrate, and control policies adapt. Such systems naturally drift across the R/D space: even therapeutic uses can accumulate discovery, and even research‐oriented operations can produce restorative or destabilizing effects. Static consent and episodic review cannot track these shifting forms of epistemic power or influence, nor the accumulation of uncertainty they produce [50]. Ethical oversight must therefore become temporally extended and situated [51], sensitive to how capabilities, vulnerabilities, and downstream dependencies develop through time.

Third, entanglement blurs the normative distinction between care and experimentation. A single operational pipeline can restore function while simultaneously generating novel insights into neural organization. Yet discovery does not guarantee recovery; it may leave function unchanged, degrade it, or unpredictably alter it. Because intention alone no longer determines what an operation does, ethical scrutiny must focus on the relationship between *purpose* and *effect* [48], and on the operation's trajectory across the R/D plane. This shifts attention from declared purpose to purpose‐bound operation: access must be calibrated to the legitimate aim of the interaction, the depth of intervention must be bounded accordingly [36], and uncertainty must be constrained in proportion to discovery.

Fourth, **epistemic intimacy** deepens silently as models improve. Epistemic intimacy can be defined as a form of closeness created by high‐resolution neural access, in which measurement touches subjective, affective, or identity‐related aspects traditionally shielded from external scrutiny. Even when raw signals remain constant, the inferential power derived from them may increase dramatically as learning algorithms refine their internal representations. Without continuous ethical attention, systems risk drifting from benign monitoring toward intrusive prediction, characterization, or control. Ethical practice, therefore, requires mechanisms that detect and constrain this **intimacy drift** [52], ensuring that precision does not silently escalate into forms of access that exceed participants’ expectations, permissions, or rights. Intimacy drift describes the gradual increase in inferential power as models learn and update, deepening epistemic intimacy even when raw data or device parameters remain unchanged. Each update of a model or recalibration of a controller modifies the operation's position in the recovery–discovery diagram, and thereby its moral profile.

In sum, entanglement‐technical, epistemic, probabilistic, and moral‐forces a paradigm shift: from ethical approval as an event to ethics as a mode of governance woven into the operation itself [50, 51]. Precision at the nanoscale demands not only improved technical control, but improved moral control‐an ongoing calibration of access, invasiveness [36], uncertainty, proportionality, and reversibility that can accompany the interface as it acts, adapts, learns, and drifts [53, 54] across the recovery–discovery landscape. This continuous mode of oversight requires infrastructures capable of absorbing errors, learning from them, and sharing lessons transparently‐an expectation already familiar in other high‐stakes technological domains, and one that will become essential here as well.

A concrete illustration of these limitations can be found in emerging applications of neural decoding and predictive modeling [55, 56]. Current ethical frameworks typically classify such systems as minimally invasive if they do not require substantial physical intervention, thereby underestimating risks associated with fine‐grained inference about mental states, preferences, or future behavior [57, 58, 59]. This can result in situations where epistemically intrusive operations remain insufficiently regulated, particularly when inference capabilities evolve over time [60, 61]. Within the framework proposed here, such cases would be addressed through continuous monitoring of epistemic depth, explicit constraints on permissible inference, and mechanisms to detect and limit drift in R/D‐position [62]. This allows ethical oversight to track not only what a system does initially, but how its capabilities expand and how this expansion affects the conditions of autonomy, privacy, and accountability [63, 64, 65]. Much like aviation safety evolved from accident‐based learning to continuous monitoring, such cases illustrate the need for adaptive ethical infrastructures capable of detecting emerging risks before harm materializes [66, 67].

## 5 The Architecture of Ethical Precision

Since nanoscale brain interfacing transforms precision into a form of **epistemic intimacy**, then ethical practice must evolve into a mode of precision governance. **Ethical precision** refers to the capacity to modulate access, influence, and inference with a granularity that matches the technological resolution of the interface. It does not seek to impede innovation, but to ensure that neural access remains proportionate, reversible, transparent, and purpose‐bound. Just as other high‐stakes technologies‐most notably the safety management system of aviation [68], learned that technical refinement must be matched by continuous monitoring, transparent incident reporting, and adaptive standards [69], precision neurotechnologies require governance architectures capable of evolving alongside their capabilities. Several such principles can translate this conceptual shift into concrete practice.

### 5.1 Calibrating Purpose in an Entangled System

Because recovery and discovery are continuous dimensions rather than discrete categories, ethical governance must respond to where an operation *is* in the recovery–discovery plane and how it *moves* through it. Anatomical measures of invasiveness no longer reliably indicate risk; operations with minimal physical footprint may nevertheless entail significant ethical exposure [36]. Rather than switching between “modes,” governance must track R/D position, detect R/D drift, and automatically adjust safeguards as an operation moves toward regions of higher discovery and greater uncertainty.

In this context, dynamic purpose calibration replaces mode separation. The intended purpose of an operation becomes an orientation‐a vector‐in the R/D plane. Governance mechanisms monitor whether the actual trajectory remains aligned with this purpose and trigger proportional escalation of oversight when the drift toward higher‐discovery zones exceeds predefined thresholds. Capability pre‐registration still plays a role, but as a declaration of the **operational envelope**, which is a concept adapted from aviation. Here it describes a defined boundary of safe and permissible system functioning that prevents ethically hazardous excursions unless explicitly justified, documented, and monitored. It determines which signals may be decoded, which inferences are permissible, and how far discovery is allowed to progress without additional authorization. Aviation's envelope‐protection systems provide the appropriate analogy: they do not disable flight modes but prevent excursions into unsafe regimes unless explicitly justified and documented [70].

### 5.2 Protecting Epistemic Intimacy

**Ethical precision** requires protecting individuals from forms of inference that exceed legitimate purpose or drift beyond ethically permissible bounds. An intimacy index can quantify epistemic depth by integrating three elements: the granularity of data, the predictive power of models, and the persistence of stored information. This index becomes a gradient trigger, scaling ethical safeguards with the level of discovery and **epistemic intimacy**. Higher positions on the discovery axis require additional consent checks, stricter constraints, or automatic firewall activation.

**Epistemic firewalls**‐algorithmic constraints that prohibit decoding affective states, personality traits, identity markers, or other morally sensitive content‐provide a structural boundary that operates regardless of technical feasibility. Where discovery is required, privacy‐preserving learning methods such as federated or split learning allow model refinement without centralizing or exposing sensitive neural data. These mechanisms parallel aviation's separation of critical subsystems: powerful analytics exist, but not every available inference is allowed to be made.

### 5.3 Enabling Continuous Ethical Oversight

Given the dynamic nature of nanoscale operations, ethical oversight must accompany the interface as it acts, adapting as the operation moves across the recovery–discovery plane. A real‐time consent dashboard can provide participants with continuous transparency regarding what is being sensed, decoded, or stimulated, along with simple controls to pause or stop the system. Deviations between what participants consented to and what the system performs‐consent drift‐should be logged and treated with the same seriousness as clinical adverse events.

To address cumulative and potentially irreversible effects, interfaces can incorporate a **reversibility budget**, a predefined limit on non‐reversible or plasticity‐inducing operations. An adaptive proportionality controller can cap system influence, tightening or relaxing constraints based on therapeutic need, participant feedback, and the operation's instantaneous position within the R/D plane. Finally, **ethics telemetry**‐machine‐readable logs of sensing events, inferential operations, stimulation parameters, model updates, and R/D position estimates can make oversight auditable, traceable, and responsive. This mirrors aviation's black‐box systems, which ensure accountability not by preventing all errors but by ensuring that no error is lost to silence. Noteworthy, these mechanisms enable continuous oversight, they also introduce new dependencies on data‐driven infrastructures.

The increasing reliance on data‐driven and potentially AI‐supported infrastructures for ethical oversight introduces a second‐order layer of ethical concern [64, 71]. Systems designed to monitor R/D‐position, detect drift, or generate ethics telemetry may themselves become sources of opacity, bias, or misplaced normative authority if their operations are not sufficiently transparent and contestable [61, 63, 72]. The automation of ethical monitoring must therefore not be conflated with the automation of ethical judgment [73]. While such systems can assist in detecting patterns, anomalies, or threshold violations beyond human perceptual capacity, they cannot determine the normative acceptability of an operation [62]. Ethical precision requires that these infrastructures remain interpretable, auditable, and subject to human oversight, with clear mechanisms for intervention, contestation, and recalibration [71, 74]. Otherwise, there is a risk that governance itself becomes technocratic‐shifting responsibility from accountable actors to opaque systems, and thereby reproducing, at a meta‐level, the very forms of epistemic and normative overreach that ethical precision seeks to constrain [60, 65, 72].

### 5.4 Safeguarding the Afterlife of Neural Data

After an operation ends, precision persists in the data it generates. Storing neural information in data trusts with fiduciary responsibilities can ensure purpose‐bound use and protect participants’ interests. Red‐teaming practices can stress‐test systems for unintended inferences or covert channels of influence, and incident registries can accumulate knowledge about ethical near‐misses across institutions, enabling sector‐wide learning [48]. In the R/D framework, these registries also track conditions under which operations drifted into high‐discovery regimes, creating an empirical basis for refining ethical thresholds and **operational envelopes**.

Together, these mechanisms constitute an emerging architecture of **Ethical Precision**. Ethical precision is the calibrated alignment of neural access and influence with legitimate purpose, proportionality, and reversibility; concerned not with acquiring more information, but with responsibly delimiting and contextualizing it. Accordingly, it represents a shift from regulating devices to governing operations, from episodic approval to continuous moral calibration. They form the foundations of a governance regime modeled not on static compliance but on adaptive safety cultures‐much like those in aviation‐designed to detect drift, learn from errors, and guide **technological precision** toward care, autonomy, and respect for mental life. Technological precision is here the refinement of measurement or intervention, such as increased resolution or reduced noise, that improves the accuracy and specificity of neural access.

## 6 Toward a Responsible Future for Intimate Precision

Nanoscale brain interfacing invites us to rethink what it means to approach the brain with precision. At this scale, precision is not only a technical capacity but a form of access that comes uncomfortably close to the foundations of neural and mental life. The explicit consideration of recovery and discovery dimensions‐between restoring what was lost and exploring what is unknown‐proves necessary to govern the new territory of **epistemic intimacy** created by these technologies [52]. Their operational entanglement dissolves familiar boundaries and demands a shift from device‐centric ethics to an ethics of continuous, operation‐sensitive oversight.

The proposals developed here aim to provide a conceptual and architectural foundation for such an ethical transformation. R/D‐position tracking, capability pre‐registration, intimacy indices, **epistemic firewalls**, **reversibility budgets**, consent dashboards, and **ethics telemetry** are not regulatory burdens but instruments of precision themselves‐tools that enable a finer calibration of access, influence, and inference. They allow innovation to unfold within boundaries that protect autonomy, privacy [36, 48], and the dignity of mental life [17], without stifling the therapeutic and scientific promise of nanoscale technologies. A culture of safety in which the mechanisms to learn from mistakes and to be perfectly open to the disclosure of accidents [75] is the minimal level of accountability we need when dealing with the precision of nano‐neurotechnology.

Crucially, this approach does not require new constitutional rights, metaphysical claims about the brain, or what might be called ***technocratic normative futurism*** frameworks that attempt to legislate hypothetical futures or proclaim oath‐like commitments detached from day‐to‐day practice [76]. While such initiatives have raised awareness, they risk codifying abstract ideals rather than supporting the concrete, situated decisions that clinicians, engineers, and researchers must make under conditions of uncertainty [36]. Instead of relying on symbolic measures or constitutional romanticism, the governance of precision neurotechnologies should draw from domains, such as aviation, where decades of experience have shown how to manage complex, sensitive, high‐stakes systems without resorting to speculative rights regimes [77].

As the fidelity of our interfaces increases, so does our responsibility to govern the proximity we create. The challenge ahead is to recognize that more precise technologies require more precise ethics‐not simply stronger regulation, but a more refined understanding of what it means to act, measure, learn, and intervene within the intimate domain of the mind. The future of precision brain interfacing will be defined not only by how deeply we can see into the brain, but by how carefully we can shape the conditions under which such seeing becomes permissible. In this spirit, the path forward lies not in reinventing our moral vocabulary, but in cultivating governance cultures capable of absorbing errors, adapting to drift, and committing‐openly and continuously‐to the responsible stewardship of neural intimacy.

## Conflicts of Interest

The author declares no conflicts of interest.
